# Positive and Negative Post Performance-Related Thoughts Predict Daily Cortisol Output in University Music Students

**DOI:** 10.3389/fpsyg.2020.585875

**Published:** 2020-11-13

**Authors:** Yoav E. Y. Haccoun, Horst Hildebrandt, Petra L. Klumb, Urs M. Nater, Patrick Gomez

**Affiliations:** ^1^ Center for Primary Care and Public Health, University of Lausanne, Lausanne, Switzerland; ^2^ Swiss University Centre for Music Physiology, Zurich University of the Arts, Zurich, Switzerland; ^3^ Swiss University Centre for Music Physiology, Basel University of the Arts, Basel, Switzerland; ^4^ Department of Psychology, University of Fribourg, Fribourg, Switzerland; ^5^ Clinical Psychology, Department of Psychology, University of Vienna, Vienna, Austria

**Keywords:** ambulatory assessment, music performance, post-performance rumination, post-performance thoughts, salivary cortisol, salivary alpha-amylase, university music students, social-evaluative stress

## Abstract

Psychophysiological research on music performance has focused on musicians’ short-term affective, cognitive, and physiological responses. Much less attention has been devoted to the investigation of musicians’ psychophysiological activity beyond the performance situation. Musicians report having both positive and negative performance-related thoughts (e.g., “My concert was good” and “I made a lot of mistakes”) for days following performances. The potential physiological implications of this post-performance cognitive processing are largely unknown. Salivary cortisol (sC) and salivary alpha-amylase (sAA) are markers of the activity of the hypothalamic-pituitary-adrenal (HPA) axis and the sympathoadrenal medullary (SAM) system, respectively. The goal of the present study was to investigate whether self-reported positive and negative post performance-related thoughts predict the daily sC output and the daily sAA activity at the between- and within-person levels during a 2-day period following a solo music performance. Seventy-two university music students collected saliva samples six times per day and reported their positive and negative performance-related thoughts for 2 days after a solo performance. We tested between-person and within-person components of positive and negative post performance-related thoughts as predictors of the diurnal area under the curve with respect to ground (AUCg) for sC and sAA while adjusting for relevant person-level and day-level variables. Negative post performance-related thoughts were positively associated with sC AUCg both at the between- and within-person levels, whereas positive post performance-related thoughts were negatively associated with sC AUCg at the between-person level. Post performance-related thoughts did not significantly predict sAA AUCg. These findings provide evidence for a relationship between affectively valenced cognitive processing of a recent music performance and the activity of the HPA axis. Although the directionality of this relationship remains to be established more conclusively, the study makes a significant contribution to the literature on the prolonged psychophysiological effects of music performance situations and more broadly of social-evaluative stressors. Integrating the topic of post-performance cognitive processing and its optimal management into performance training programs would likely have positive effects on music students.

## Introduction

Performing publically at a professional level is a demanding activity for many musicians, often associated with intense short-term emotional, cognitive, and physiological responses (e.g., Pilger et al., 2014; Studer et al., 2014; Oudejans et al., 2017). There is some evidence that the psychophysiological effects of music performances can extend well beyond the performance situation itself. For instance, some musicians report experiencing increased anxiety days or even weeks before performing (Van Kemenade et al., 1995; Hildebrandt et al., 2012). Recently, Nielsen et al. (2018) showed that university music students report having performance-related thoughts for at least 2 days following a solo performance. The content of these thoughts can be positively valenced (e.g., “My concert was good”) or negatively valenced (e.g., “I made a lot of mistakes”). This mental reviewing of a performance situation can be understood within the concept of post-event rumination (also called post-event processing and post-mortem thinking; Watkins, 2008). Kashdan and Roberts (2007, pp. 285–286) have defined post-event rumination as “repetitive thoughts about subjective experiences during a recent social interaction, including self-appraisals and external evaluations of partners and other details involving the event.” Post-event rumination has been mainly conceptualized and investigated within the social anxiety literature (Clark and Wells, 1995; Rapee and Heimberg, 1997). Compared to non-socially anxious individuals, socially anxious individuals have been consistently found to report more negatively valenced thoughts following a speech or conversation (Mellings and Alden, 2000; Edwards et al., 2003; Abbott and Rapee, 2004; Perini et al., 2006; Dannahy and Stopa, 2007; Kocovski et al., 2011). With regard to positively valenced thoughts, findings have been mixed with three studies showing no significant effects of social anxiety (Edwards et al., 2003; Abbott and Rapee, 2004; Dannahy and Stopa, 2007) and one study reporting significantly fewer positively valenced thoughts among socially anxious participants than among non-socially anxious participants (Kocovski et al., 2011). In the 2 days following a solo performance, music students with high levels of music performance anxiety reported more negatively valenced thoughts and fewer positively valenced thoughts than music students with low levels of music performance anxiety did (Nielsen et al., 2018).

There is some evidence that negatively valenced post-event rumination predicts negative outcomes. These include increased anticipatory anxiety (Brozovich and Heimberg, 2013; Blackie and Kocovski, 2016), more negative effect (Kashdan and Roberts, 2007), more socially anxious interpretations of ambiguous social situations (Brozovich and Heimberg, 2013), increased recall of negative self-related information, and negative self-judgments when anticipating further social interactions (Mellings and Alden, 2000). Whether positively and negatively valenced post-event cognitions differently affect physiological processes such as the activity of the hypothalamic-pituitary-adrenal (HPA) axis and the activity of the sympathoadrenal medullary (SAM) system is largely unknown.

The HPA axis is a central regulatory system implicated in the organism’s reaction to stressors (Kaltsas and Chrousos, 2007; Nater et al., 2013). The catabolic hormone cortisol is secreted from the adrenal gland into the blood stream in response to the adrenocorticotropic hormone (Reisch et al., 2005). Cortisol regulates many processes such as immune system activity and glucose metabolism (Sapolsky et al., 2000). Following a circadian rhythm, cortisol exhibits the highest levels after awakening, followed by a decline throughout the afternoon and evening (Hucklebridge et al., 2005). In response to social-evaluative situations such as music performances, most people acutely display increased salivary cortisol (sC) secretion (e.g., Dickerson and Kemeny, 2004; Pilger et al., 2014; Aufegger and Wasley, 2018). These responses can be prolonged for hours before and after actual exposure to social-evaluative stressors, thus increasing the daily cortisol output (Rohleder et al., 2007; Wetherell et al., 2015; Gomez et al., 2018).

The SAM system is a second important regulatory system involved in the response to stressors. It is primarily regulated by the locus coeruleus-norepinephrine system in the brainstem (Dunn et al., 2004; Kaltsas and Chrousos, 2007). In response to stressors, the adrenal gland releases norepinephrine and epinephrine into the blood stream. A downside of these parameters is the need for blood taking. Salivary alpha-amylase (sAA), an enzyme mainly secreted from the acinar cells of the salivary glands, has gained interest over the last 15 years as a marker for the activity of the SAM system as it can be measured by sampling saliva (Warren et al., 2017; Ali and Nater, 2020). The activity of sAA is low in the morning and steadily increases over the course of the day, typically reaching its peak in the late afternoon (Nater et al., 2007). In healthy individuals, social-evaluative stressors induce increased sAA activity (e.g., Nater et al., 2007; Filaire et al., 2010; Thoma et al., 2012), and this increase can be prolonged well beyond the exposure to the stressor (Gomez et al., 2018).

The valence of the thought content has been highlighted as a key determinant for understanding the consequences of thought processes (Watkins, 2008). The literature on the relationship between affectively negative thoughts and physiological activity has been much more developed than the literature on the relationship between affectively positive thoughts and physiological activity. One prominent theory in the context of negatively valenced thoughts is the perseverative cognition hypothesis (Brosschot, 2010). According to this hypothesis, perseverative cognition, defined as “repetitive or sustained activation of cognitive representations of past stressful events or feared events in the future” (Brosschot, 2010, p. 407), leads to prolonged or repeated neuroendocrine activation, potentially contributing to the development of disease. Empirical evidence supports this hypothesis. Perseverative cognition is significantly associated with higher blood pressure, higher heart rate, lower heart rate variability, and higher cortisol levels (Ottaviani et al., 2016) and significantly predicts cardiovascular health problems in the long-term (Kubzansky et al., 1997; Holman et al., 2008). One study has also provided initial evidence for a positive association between perseverative cognition and daily sAA activity (Gomez et al., 2018).

Associations between positive psychological states (broadly defined to include positive affect, positive cognitions, and other resources such as optimism, self-esteem, and resilience; cf. Low et al., 2011; Kubzansky et al., 2015) and activity of the HPA axis have been reported. As reviewed by Chida and Hamer (2008) and Low et al. (2011), there is evidence that positive psychological states are associated with lower cortisol levels. We are not aware of research specifically investigating the link between positively valenced thoughts and indicators of the activity of the SAM system, including sAA.

The goal of the present study was to investigate whether positive and negative performance-related thoughts predicted the daily sC output and the daily sAA activity at the between- and within-person levels in university music students during the first 2 days following a solo performance. Drawing from the literature introduced above, we hypothesized that both positive and negative post-performance thoughts would be significant predictors of daily sC output and daily sAA activity. Specifically, we hypothesized that negatively valenced thoughts would be significantly associated with higher daily sC output and with higher daily sAA activity and that positively valenced thoughts would be significantly associated with lower daily sC output and with lower daily sAA activity.

## Materials and Methods

### Participants

The participants were students at Swiss music universities. Prospective participants were administered an entry questionnaire and excluded if they had any of the following: any known endocrine or cardiovascular disease; use of psychoactive drugs or any medication affecting the biological systems under study; being pregnant; lactating; wearing a pacemaker; working night shifts; major depression syndrome, bulimia, binge eating disorder, and alcohol abuse as assessed using the Patient Health Questionnaire (Spitzer et al., 2000). We excluded 18 students based on these criteria leaving a final sample of 72 participants (mean age = 22.7, *SD* = 3.0; 65% female). All participants gave their written informed consent to participate in the study and were remunerated 500 Swiss francs. This sample has been utilized previously to address different research questions (Gomez et al., 2018; Nielsen et al., 2018; Studer et al., 2019).

### Procedure of the Ambulatory Assessment

We tested the participants during a 7-day period that was composed of 4 pre-concert days, 1 concert day, and 2 post-concert days. The present study focuses on the 2 post-concert days. On the concert day, students performed solo one or more self-selected musical pieces (5–10 min in total) before an audience of 10–15 persons (*M* = 12.6, *SD* = 1.4) who were introduced to the students as music connoisseurs. The performances took place in small concert halls located in the music schools. The concerts started between 3 and 6 PM. Students performed once and had no other solo performances during the assessment period.

Participants collected six saliva samples per day (immediately after awakening, 30 min after awakening, 11 AM, 2 PM, 6 PM, 9 PM). They also filled in questionnaires on each occasion except at the first time point. Prior to the start of the assessment period, the experimenter trained the participants on how to collect saliva and fill in the questionnaires handling a pre-programed iPod® touch 5 (iDialog Pad, Gerhard Mutz, Cologne University, Germany). Sampling times were automatically registered on the iPod.

### Measures

We first describe the measures of main interest (post performance-related thoughts, sC, and sAA) and then control variables.

#### Post Performance-Related Positive and Negative Thoughts

With the last questionnaire of each of the 2 post-concert days, participants reported their positive and negative performance-related thoughts. We assessed these thoughts with the 23-item Post-Music Performance Thoughts Questionnaire, which is an adaptation of the Post-event Rumination Questionnaire originally used to assess thoughts after a speech (Abbott and Rapee, 2004; Perini et al., 2006). The questionnaire consists of nine positive items (e.g., “My concert was good”) and 14 negative items (e.g., “I made a lot of mistakes”). Participants were required to report to what extent they had each thought since the end of the concert (first post-concert day) and during the last 24 h (second post-concert day) using a five-point Likert scale ranging from 0 “not at all” to 4 “extremely.” The total score of the positive items ranges from 0 to 36, whereas the total score of the negative items ranges from 0 to 56, with higher scores corresponding to more positive and negative thoughts, respectively. Internal consistencies of both scales in the present study were excellent (Cronbach’s alphas >0.90).

#### Salivary Cortisol and Salivary Alpha-Amylase

We obtained saliva samples *via* a passive drooling method using SaliCaps® (IBL, Hamburg, Germany). We instructed the participants to rinse their mouth with water whenever possible and to swallow or spit the saliva currently in their mouth before accumulating saliva for 2 min in their mouth (using a timer within iDialog Pad) and finally transferring all saliva into the tubes. Participants collected the first sample immediately after awakening when still lying in bed. We required them to avoid eating, drinking, smoking, brushing their teeth and performing intense physical activity between the first and second sample and to avoid eating, smoking, brushing their teeth, and drinking caffeinated and alcoholic beverages and fruit juices for at least 30 min prior to collection of the other four samples.

Samples were stored during the assessment period in the participants’ fridges and then kept in a freezer at −20°C in our lab before being sent on dry ice to the Biochemical Laboratory of the Department of Clinical Biopsychology, University of Marburg, Germany, where they were again stored at −20°C until biochemical analyses. We measured free sC concentrations using a commercially available enzyme-linked immunosorbent assay (IBL, Hamburg, Germany) and sAA activity using an in-house kinetic colorimetric test with reagents obtained from Roche Diagnostics (Mannheim, Germany). Intra- and inter-assay coefficients of variation were 8.4 and 10.3 for sC and 5.4 and 14.3 for sAA, respectively. We analyzed the diurnal area under the curve with respect to the ground (sC AUCg and sAA AUCg), which was calculated *via* a trapezoidal function using all available samples (Pruessner et al., 2003).

#### Anxieties and Depressive Symptoms

Before the ambulatory assessment, participants completed online questionnaires assessing music performance anxiety, social anxiety, and depressive symptoms, all of which are potential confounding variables for our hypotheses because they can affect sC output and sAA activity (Burke et al., 2005; Van Veen et al., 2008; Crişan et al., 2016; Gomez et al., 2018).

We measured music performance anxiety, conceptualized as the person’s general tendency to experience anxiety in music performance situations, with the 20-item state form of the State-Trait Anxiety Inventory (Spielberger, 1983). Because the performance setting affects the anxiety level, we adapted the instructions to music performance situations and asked the participants to answer each item by referring to how they generally feel when performing solo. Each item (e.g., “I am tense”) is rated on a 4-point Likert scale (1 “not at all” to 4 “very much so”). The score ranges from 20 to 80 with higher scores corresponding to more severe music performance anxiety. Cronbach’s alpha of this scale in the present study was 0.93.

We measured social anxiety, conceptualized as the person’s tendency to experience anxiety in social and performance situations in general, with the self-report version of the 24-item Liebowitz Social Anxiety Scale (Fresco et al., 2001). Each item (e.g., “Calling someone you do not know very well”) is rated in terms of experienced fear on a scale from 0 (none) to 3 (severe) and in terms of avoidance behavior on a scale from 0 (never) to 3 (usually). The score ranges from 0 to 144 with higher scores corresponding to more severe social anxiety. Cronbach’s alpha of this scale in the present study was 0.94.

We assessed depressive symptoms with the 21-item Beck Depression Inventory (Beck et al., 1996). Each item contains four sentences, coded from 0 (less close to depression, e.g., “I do not feel sad”) to 3 (closest to depression, e.g., “I am so sad or unhappy that I can’t stand it”). The score ranges from 0 to 63 with higher scores corresponding to more severe depressive symptoms. Cronbach’s alpha of this scale in the present study was 0.78.

#### Wake-Up Time

Participants reported their wake-up time every day. We included this time as control variable because the computation of the AUCg depends on the time interval (Pruessner et al., 2003).

#### Stressful Events

On each sampling occasion, except immediately after awakening, participants reported the number of stressful events experienced since the last assessment time/since waking up. We provided the following definition of a stressful event based on Verkuil et al. (2012): “Stressful events are minor and major events that have made you feel tense, irritated, angry, sad, disappointed or negative in any other way.” We computed the daily sum of all stressful events and used this in our model in order to control for this potential confounding effect (Van Eck et al., 1996).

#### Bio-Behavioral Measures

On each sampling occasion, except immediately after awakening, participants indicated the number of units of caffeinated beverages, alcoholic beverages, and tobacco products consumed since the last assessment time/since waking up. We included these measures as control variables because they can affect sC output and sAA activity (Strahler et al., 2017).

### Data Processing and Statistical Analyses

For the computation of AUCg, we required the first saliva sample to be collected within 10 min of the self-reported wake-up time and the 30-min post-wake saliva sample to be collected within 15 min of the expected time. We additionally required the first, second, and sixth saliva sample to be available (Out et al., 2013) and the sixth saliva sample to be collected between 8 and 10 PM. When values were available for other time points, they were also included in the computation (Out et al., 2013). The sC AUCg scores were computed on average from 5.8 (*SD* = 0.4) sC values per day. The sAA AUCg scores were computed on average from 5.7 (*SD* = 0.5) sAA values per day. AUCg scores were log transformed to better approximate normal distribution.

We performed all statistical analyses using STATA version 16.0 for Windows (Stata Statistical Software; StataCorp LP, College Station, TX). The alpha level was set at 0.05. We fitted two-level linear mixed models with restricted maximum likelihood estimation for analysis of sC AUCg and sAA AUCg as outcomes. We decomposed the main predictors into their within-person component (i.e., within-person centered positive and negative post performance-related thoughts representing deviations from an individual’s average during the 2 post-concert days) and between-person component (i.e., grand-mean centered positive and negative post performance-related thoughts representing deviations from an individual’s average score relative to the sample average). For sC AUCg and sAA AUCg separately, we tested a model with the between- and within-person components of the positive and negative post performance-related thoughts as predictors of main interest. We also included in the models the control variables music performance anxiety, social anxiety, depressive symptoms, age, gender, hormonal contraception (yes/no), daily wake-up time, daily number of stressful events and daily consumption of caffeine, alcohol, and tobacco. Participants performed the concert on different days of the week and different periods of the year, factors that can affect sC concentration and sAA activity (Kunz-Ebrecht et al., 2004; Persson et al., 2008). Therefore, day of the week (Sunday, etc.) and season (spring, etc.) were also included as control variables. We used likelihood ratio tests for model selection (West et al., 2015). For both sC AUCg and sAA AUCg, the estimated model included a random intercept for each participant and homogenous residual variance structure. For sC AUC, we also specified a random slope for wake-up time with independent random effects covariance structure as this improved the model significantly. Diagnostics for residuals and random effects showed that distributional assumptions were met for all models implying satisfactory model specification.

## Results

### Descriptive Statistics

Table 1 gives descriptive statistics for the main predictors, the control variables, and the outcome variables.

**Table 1:** Descriptive statistics of predictors and outcome variables.

	N	*M* (*SD*)	Min-max
Positive thoughts within-person		3.5 (4.4)	0–19
Positive thoughts between-person		8.1 (7.3)	0–35
Negative thoughts within-person		3.2 (3.1)	0–14
Negative thoughts between-person		9.6 (10.7)	0–48.5
Wake-up time (hrs:min)		8:14 (1:22)	5:15–12:55
Number of caffeinated beverages per day		1.0 (1.1)	0–5
Number of alcoholic beverages per day		0.3 (0.6)	0–3
Number of cigarettes per day		0.6 (1.7)	0–10
Number of stressful events per day		1.2 (2.1)	0–12
Music performance anxiety		49.6 (11.7)	27–73
Social anxiety		34.2 (21.2)	4–98
Depressive symptoms		6.2 (5.0)	0–21
Age (years)		22.7 (3.0)	18–30
Gender			
Men	25		
Women	47		
Hormonal contraception			
Yes	24		
No	23		
sC AUCg (log-transformed)		9.2 (0.5)	8.1–10.7
sAA AUCg (log-transformed)		11.0 (0.9)	9.0–12.8

### Salivary Cortisol

Table 2 gives the model for sC AUCg. Positive post performance-related thoughts were significant predictors of sC AUCg at the between-person level (negative estimated coefficient). Negative post performance-related thoughts were significant predictors of sC AUCg both at the between- and within-person levels (positive estimated coefficients).

**Table 2:** Estimated linear mixed models for sC AUCg and sAA AUCg.

	sC AUCg				sAA AUCg			
	Coeff.	*SE*	*χ^2^*	*p*	Coeff.	*SE*	*χ^2^*	*p*
Intercept	9.1	0.1			12.4	0.4		
Positive thoughts within-person	−1.1	1.9	0.36	0.54	−1.0	3.8	0.07	0.79
Positive thoughts between-person	**−1.7**	**0.8**	**5.29**	**0.021**	2.5	2.0	1.56	0.21
Negative thoughts within-person	**3.3**	**1.4**	**5.38**	**0.021**	3.9	4.5	0.72	0.39
Negative thoughts between-person	**2.0**	**0.5**	**19.18**	**<0.001**	−0.7	1.4	0.26	0.61
Wake-up time	−20.4	5.3	19.71	<0.001	−3.2	9.2	0.14	0.71
Number of caffeinated beverages	9.1	4.8	3.46	0.063	4.8	14.4	0.12	0.72
Number of alcoholic beverages	2.0	7.2	0.08	0.77	−33.6	24.1	3.61	0.058
Number of cigarettes	1.1	2.4	0.20	0.65	−4.1	6.2	0.48	0.48
Number of stressful events	3.4	1.5	4.84	0.028	−8.6	5.2	3.17	0.076
Music performance anxiety	−4.2	0.5	68.39	<0.001	3.9	1.6	6.05	0.014
Social anxiety	1.1	0.3	19.89	<0.001	0.1	0.7	0.02	0.90
Depressive symptoms	1.3	0.9	1.85	0.17	3.4	2.7	1.54	0.21
Age	0.4	1.4	0.08	0.77	−3.0	4.2	0.55	0.46
Gender	19.6	12.1	2.46	0.11	−39.3	39.5	2.25	0.13
Hormonal contraception	17.9	11.4	2.34	0.12	−34.8	38.8	1.69	0.19
Day of the week			16.32	0.006			12.37	0.030
*Tuesday*	−16.5	17.5			−48.2	66.7		
*Wednesday*	−7.9	14.1			−58.8	54.5		
*Thursday*	−30.5	14.0			−72.0	49.1		
*Friday*	−32.7	15.2			−68.0	51.6		
*Saturday*	−0.5	9.0			−17.1	28.9		
Season			9.09	0.028			9.23	0.026
*Summer*	−5.9	19.3			−72.8	73.2		
*Fall*	31.5	13.4			43.5	42.3		
*Winter*	33.4	12.3			−27.7	41.7		

Reference categories for categorical predictors were as follows: gender: men; hormonal contraception: naturally cycling women; day of the week: Sunday; season: spring. For continuous predictors, coefficients express the change in the outcome measure per unit. Units are as follows: positive and negative thoughts within- and between-person, music performance anxiety, social anxiety, depressive symptoms: 1 point on the corresponding scales; wake-up time: 1 h; number of caffeinated beverages: 1 beverage; number of alcoholic beverages: 1 beverage; number of cigarettes: 1 cigarette; number of stressful events: 1 event; age: 1 year; Coeff. = estimated coefficient; *SE* = standard error. Degrees of freedom for *χ* ^2^ are five for day of the week, three for season, and one for all other predictors. Statistically significant results for the predictors positive and negative post performance-related thoughts are marked in bold.

### Salivary Alpha-Amylase

Between- and within-person components of positive and negative post performance-related thoughts were not significant predictors of sAA AUCg (see Table 2).

## Discussion

In line with our hypotheses, we found that negative post performance-related thoughts were significant predictors of daily sC output both at the between- and within-person levels. Participants who reported more negative performance-related thoughts exhibited significantly higher daily sC output during the 2 post-concert days than participants who reported fewer negative performance-related thoughts. At the intra-individual level, we found that an increase in negative post performance-related thoughts from 1 day to the other was significantly associated with an increase in daily sC output. These findings are in accordance with the broader literature on perseverative cognition and physiological activation, which suggests a positive relationship between perseverative cognition and cortisol secretion (Ottaviani et al., 2016). The negative post performance-related thoughts assessed in the present study can be conceived as a specific form of perseverative cognition. According to the perseverative cognition hypothesis, perseverative cognition about a stressor such as a public music performance prolongs the psychophysiological stress response (Brosschot, 2010). Our findings are consistent with this hypothesis.

Confirming our expectations, we also found that positive post performance-related thoughts were significant predictors of daily sC output at the between-person level. Participants who reported more positive performance-related thoughts exhibited significantly lower daily sC output during the 2 post-concert days than participants who reported fewer positive performance-related thoughts. To the best of our knowledge, this is the first study to show that between-person differences in positively valenced cognitions are significantly associated with individuals’ daily sC output. This finding is in accordance with and extends the literature on the biology of positive psychological functioning, which suggests that a broad range of positive psychological states and traits such as positive effect, benefit finding, active coping, and optimism are associated with lower cortisol levels (Chida and Hamer, 2008; Low et al., 2011). Moreover, the present finding suggests that at the between-person level positive post performance-related thoughts might have unique implications for the activity of the HPA axis beyond the effects of negative post performance-related thoughts. This finding parallels results from studies providing evidence of a relationship between positive psychological states (e.g., trait positive effect) and physiological activity independent of the relationship between negative psychological states (e.g., trait negative effect) and physiological activity (Marsland et al., 2006; Prather et al., 2007; Brydon et al., 2009).

The significant associations between post performance-related thoughts and sC output can be interpreted from the perspective of psychophysiological recovery from social-evaluative stressors. Although the directionality of these relationships needs to be determined more conclusively, our findings are in accordance with an interpretation that negative post performance-related thoughts might contribute to a slower HPA axis recovery, whereas positive post performance-related thoughts might contribute to a more rapid HPA axis recovery (Fredrickson and Levenson, 1998; Ong et al., 2006). The biological response to stressors can impose long-term damage if it fails to shut down frequently or chronically (i.e., poor post-stress recovery; Chida and Steptoe, 2010; Low et al., 2011; McEwen, 2016). Thus, considering that university music students perform regularly, the present findings about the link between post-performance processing and cortisol output are potentially relevant to their long-term well-being and health.

Day-to-day variations in positive post performance-related thoughts were not significant predictors of daily sC output, even though the estimated relationship was in the expected direction. Lack of variance in the within-person component of positive post performance-related thoughts is unlikely to be an explanation for this null finding, given that it was in the same range as the within-person component of negative post performance-related thoughts for which we obtained a significant effect. Although we adjusted for several day-level variables in our analyses (e.g., number of stressful events), we cannot exclude that we failed to assess important day-level confounders of positive post performance-related thoughts.

Contrary to our hypotheses, we did not find any significant relationships between post performance-related thoughts and daily sAA activity. This finding would be in agreement with an interpretation that post performance-related thoughts do not significantly affect the activity of the SAM system. This result partially contrasts with an analysis showing that the within-person component of concert-related perseverative cognition significantly predicted daily sAA activity (Gomez et al., 2018). Possible reasons for this discrepancy relate to differences in the measurement of performance-related cognition.

In the analyses by Gomez et al. (2018), participants reported the duration of thoughts/images about problems; preoccupations or any negative events, experiences or situations from the past, the present, or the future. They further estimated how many minutes of these thoughts/images were about the concert.

The participants were asked to estimate these durations during the 4 days before the concert, on the day of the concert, and during the 2 days after the concert. The intra-individual positive association between the duration of concert-related perseverative cognition and daily sAA activity was significant when all 7 days were analyzed together but not when we limited the analysis to the 2 post-concert days (*p* = 0.62).

A meta-analysis revealed that positive psychological states were associated with lower cortisol reactivity but were not related to changes in circulating catecholamines (Chida and Hamer, 2008). As noted by these authors, the HPA axis and the SAM system are regulated by interconnected yet distinct brain circuits (Kaltsas and Chrousos, 2007). Our findings would be consistent with the idea that affectively valenced cognitive processing affects the activity of the HPA axis and of the SAM system differently. It is important to note that sAA is a surrogate indicator of the activity of the SAM system, contrary to cortisol, which is a more direct indicator of the activity of the HPA axis. Before drawing any conclusions about the relationship between the activity of the SAM system and affectively valenced cognitions, research incorporating direct SAM measures is needed.

We discuss some limitations of this study and directions for future investigations. The analyses of the associations between positive and negative post performance-related thoughts and the salivary biomarkers were limited to 2 post-concert days. Cognitive reviewing of a performance situation extends beyond 2 days after its occurrence (Dannahy and Stopa, 2007). Analyzing a longer post-performance period would allow testing the association between post performance cognitive processing and biological activity with a larger number of data points.

The participants of this study reported to have on average nine solo performances per year (ranging from 3 to 23), making the findings of this study highly relevant to this population. It would be important to investigate whether the findings of the present study also apply to other performance situations, some of which are frequent among music students, such as lessons with the teacher, rehearsals, and ensemble performances.

In the present study, we considered two aspects of post performance-related thoughts, their affective valence (positive/negative), and their amount (from “not at all” to “extremely”). Research has highlighted a number of features of thought processes as potentially important for understanding their consequences. These characteristics include repetitiveness, intrusiveness, difficulties with disengagement (Ehring et al., 2011), level of construal (abstract vs. concrete processing; Watkins, 2008), and whether cognitions include vivid mental images (Brozovich and Heimberg, 2013). For instance, socially anxious individuals who engaged in post-event processing about a past speech incorporating vivid imagery displayed greater anxiety prior to a subsequent speech task than socially anxious individuals who engaged in verbal post-event processing (Brozovich and Heimberg, 2013). Investigating the implications of these thought properties for biological processes is an important area to pursue.

The post performance-related thoughts were measured with a self-report questionnaire. Consequently, we were unable to capture unconscious cognitive activity, which may play an important role in biological functioning (Brosschot et al., 2010, 2017).

Our biological measures were sC and sAA. Extending the present investigation to other physiological parameters such as cardiovascular and inflammatory ones would allow for greater insight into the possible biological effects of post-performance processing by providing a wider biological systems approach (Low et al., 2011; Ottaviani et al., 2017).

A strength of the present study is that we included several important control variables in our analyses. One factor that we did not consider is participants’ mode of awakening (spontaneous vs. externally woken). Although there is no evidence of an effect of mode of awakening on sC output (Stalder et al., 2016), sAA was found to be significantly lower in the first hour after awakening in participants who woke up spontaneously compared with participants who woke up with an alarm clock (Nater et al., 2007). Future ambulatory assessment studies may refine analyses by taking into account mode of awakening in addition to the other factors.

We analyzed positive and negative post performance-related thoughts as predictors of daily sC output and daily sAA activity based on evidence suggesting that affectively valenced cognitions affect physiological activity (Ottaviani et al., 2016). Yet, our cross-sectional analytical approach prevents us from making any inferences about causality. Moreover, bidirectionality should not be excluded (Wirth and Gaffey, 2013; Hoyt et al., 2016). Experimental studies are needed to disentangle the relationship between post performance-related thoughts and physiological activity.

In conclusion, we have shown that during a 2-day period following a solo performance, music students reporting more negative performance-related thoughts exhibited significantly higher daily sC output than music students reporting fewer negative performance-related thoughts. Furthermore, students reporting more positive performance-related thoughts had significantly lower daily sC output than music students reporting fewer positive performance-related thoughts. At the within-person level, negative performance-related thoughts were significantly associated with higher daily sC output, whereas positive performance-related thoughts did not significantly predict differences in daily sC output from 1 day to the other. We found no significant relationships between post performance-related thoughts and daily sAA activity. These results emerged when adjusting for several relevant person- and day-level variables. These findings suggest that both positive and negative thoughts about a recent solo music performance may significantly affect the activity of the HPA axis and more generally point to the importance of taking a broader temporal perspective when studying the psychophysiological effects of music performances. Integrating the topic of post-performance cognitive processing and its optimal management into performance training programs would likely have positive effects on music students (Hildebrandt, 2009).

## Data Availability Statement

The raw data supporting the conclusions of this article will be made available by the authors, without undue reservation.

## Ethics Statement

The studies involving human participants were reviewed and approved by Ethics Committee of the canton of Vaud, Switzerland. The patients/participants provided their written informed consent to participate in this study.

## Author Contributions

All authors listed have made a substantial, direct and intellectual contribution to the present work and approved it for publication.

### Conflict of Interest

The authors declare that the research was conducted in the absence of any commercial or financial relationships that could be construed as a potential conflict of interest.
